# Luminescence Properties and Judd–Ofelt Analysis of Various ErF_3_ Concentration-Doped BaF_2_ Crystals

**DOI:** 10.3390/ma14154221

**Published:** 2021-07-28

**Authors:** Andrei Racu, Marius Stef, Gabriel Buse, Irina Nicoara, Daniel Vizman

**Affiliations:** 1 Faculty of Physics, West University of Timisoara, 4 Bd.V. Parvan, 300223 Timisoara, Romania; andrei.racu83@e-uvt.ro (A.R.); marius.stef@e-uvt.ro (M.S.); nicoara1@yahoo.com (I.N.); daniel.vizman@e-uvt.ro (D.V.); 2 National Institute of Research & Development for Electrochemistry and Condensed Matter—INCEMC Timisoara, 144 Aurel Păunescu-Podeanu Street, 300569 Timisoara, Romania

**Keywords:** barium fluoride crystal, Er doping, optical properties, Judd–Ofelt analysis, luminescence

## Abstract

The influence of erbium ion concentration on the optical properties of BaF_2_:ErF_3_ crystals was investigated. Four ErF_3_ concentration (0.05, 0.08, 0.15 and 0.5 mol% ErF_3_)-doped BaF_2_ crystals were obtained using the Bridgman technique. Room temperature optical absorption in the 250–850 nm spectral range was measured, and the photoluminescence (PL) and decay times were also investigated. The Judd–Ofelt (JO) approximation was used, taking into account four absorption peaks (at 377, 519, 653 and 802 nm). The JO intensity parameters, Ω_t_ (*t* = 2, 4, 6), were calculated. The influence of the ErF_3_ concentration on the JO parameters, branching ratio, radiative transition probability and radiative lifetime were studied. The obtained results were compared with measured values and with those reported in the literature. Under excitation at 380 nm, the well-known green (539 nm) and red (668 nm) emissions were obtained. The calculated and experimental radiative lifetimes were in millisecond range for green and red emissions. The intensity of the PL spectra varied with the Er^3+^ ion concentration. The emission intensity increased linearly or exponentially, depending on the ErF_3_ concentration. Under excitation at 290 nm, separate to the green and red emissions, a new UV emission band (at 321 nm) was obtained. Other research has not reported the UV emission or the influence of ErF_3_ concentration on emission behavior.

## 1. Introduction

Doped fluoride (MeF_2_: Me = Ca, Sr, Ba) crystals have been widely studied in order to find new scintillator and laser materials. Rare-earth (RE) ion-doped fluorides (MeF_2_), due to their optical properties, have been studied for various applications [1]. Pure BaF_2_ is a good scintillator for elementary particle and *γ*-ray detection. The optical and luminescence behavior of RE:BaF_2_ crystals are less investigated than the other fluorides. To keep the charge neutrality of the MeF_2_ lattice, the RE^3+^ ions dissolved in MeF_2_ need charge compensation. As a result, isolated centers, such as *O_h_*, *C_4v_* and *C_3v_*, and clusters will appear [2]. Using a site-selective laser excitation method, Wells [3] proved that, in the case of BaF_2_, the dominant center has C_3v_ symmetry for low ErF_3_ concentrations (<0.1 mol%). The dielectric relaxation studies [4,5,6,7] also pointed out that only trigonal *C_3v_* (*NNN*) centers are created.

The search for laser materials with UV emissions is a current necessity. In the context of the Covid pandemic, UV radiation is important for air purification. It has also been proved that UV radiation may be used for tissue treatments, for skin diseases, such as lymphoma, vitiligo and psoriasis [8].

Our preliminary luminescence experiments on a 0.2 mol% ErF_3_:BaF_2_ sample showed emission in the near-UV domain [9]. The Er^3+^:MeF_2_ crystals were studied for their properties that are good for cascade excitation [10,11,12]. Under excitation at 805 nm, Patel et al. [13] demonstrated that BaF_2_:Er^3+^ crystals generate red, green and UV emissions more efficiently than the CaF_2_:Er^3+^ crystals. Wojtowicz [14] identified emission bands in VIS, UV and VUV spectral regions by excitation in the VUV domain. The green and red emissions observed by Zhang et al. [15] in Er^3+^:BaF_2_ crystals were very weak in comparison with those obtained for Er^3+^:BaCl_2_ crystals. The emission bands were obtained by 808 nm excitation. The green emission is the strongest by 976 nm excitation (the ^2^H_1/2,_ ^4^S_11/2_ → ^4^I_15/2_ transition). Bitam et al. [16] investigated the luminescence properties of BaF_2_: 2 mol% ErF_3_ crystals. They observed a red emission, 200 times weaker than the green emission, under excitation at 378 nm. The emission spectrum of Tb^3+^-doped BaF_2_ observed by Witkowski and Wojtowicz [17] consisted of blue and green emissions. Orlovskii et al. [18] investigated BaF_2_:(0.35–1.3 at.% HoF_3_ and 0.3–2.1 at.% TmF_3_) crystals, using sensitization of Ho^3+^ fluorescence by Tm^3+^. By excitation at 980 nm, two emissions were reported by [19] in SrGe_4_O_9_:Er^3+^, Yb^3+^ phosphors. The influence of Yb^3+^ concentration on the green (551 nm) and red (662 nm) emissions were studied. As the YbF_3_ concentration exceeded 5 at.%, the red band became more intense than the green band. The Near Infrared (NIR) emission, due to Er^3+^ ions, increased five times as the YbF_3_ concentration increased. The strongest upconversion was obtained for the S8 at.% YbF_3_-doped sample.

Only a few papers that analyze the luminescence of the RE:BaF_2_ crystals, especially doped with Er^3+^ ions, can be found in the literature. The reported investigations refer to crystals doped with a high RE concentration. The influence of Er^3+^ ion concentration in BaF_2_ on the luminescence properties of this material has not yet been reported. The Judd–Ofelt (JO) semi-empirical analysis [20,21] allows the calculation of the transition probabilities, branching-ratios and radiative lifetimes in RE-doped materials using only the optical absorption spectra. Bitam et al. [16], using the JO method, reported the calculated and experimental radiative lifetime of Er^3+^ states and the quantum efficiency in the case of 2 mol% Er^3+^-doped BaF_2_. The JO intensity parameters Ω_i_ (i = 2, 3, 4) for Er^3+^ ions of *f-f* transitions and comparative analysis of the calculated and measured lifetimes were studied by Stef et al. [9] for BaF_2_: 0.2 mol% ErF_3_ and by Preda et al. [22] for Er^3+^:CaF_2_. 

The goal of this paper is to investigate the optical and luminescence behavior of low ErF_3_ concentration (0.05–0.5 mol%)-doped BaF_2_ crystals. To achieve these objectives, optical absorption and photoluminescence (PL) measurements were taken, and the Judd–Ofelt (JO) model was used to obtain information about the luminescence properties of the Er^3+^:BaF_2_ crystals. The obtained theoretical values were compared with the experimental results. We focused on the influence of ErF_3_ concentration on the optical and luminescence behavior. To our knowledge, no other report on this behavior can be found in the literature. 

## 2. Materials and Methods 

**Figure 1:**
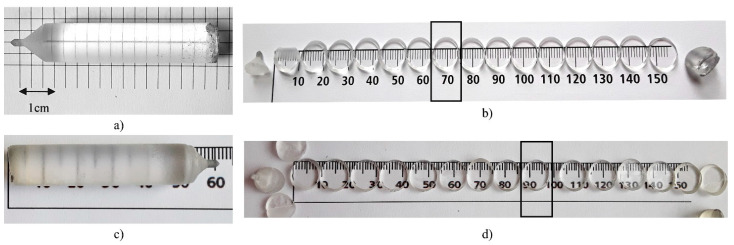
(**a**) As-grown BaF_2_: 0.08 mol% ErF_3_ crystal and (**b**) cleaved samples; the studied slice 7 is indicated. (**c**) As-grown BaF_2_: 0.15 mol% ErF_3_ crystal and (**d**) cleaved samples.

In order to obtain the four ErF_3_-doped BaF_2_ crystals, the Stockbarger–Bridgman method was used. Crushed BaF_2_ optical UV-VIS windows (Crystran Ltd. UK) were used as raw material. We aimed to investigate the properties of BaF_2_ crystals doped with low ErF_3_ concentrations. Therefore, first we added 0.05 mol% ErF_3_ to the BaF_2_ powder and chose a step of 0.05 mol% ErF_3_. The ErF_3_ came from Merck (99.99%). Next, we added 0.1, 0.15 mol% ErF_3_ and a concentration of 0.5 mol% ErF_3_, ten times higher than the lowest. Checking the optical absorption spectrum of the 0.1 mol% ErF_3_ sample and comparing it with the other samples, we found that the concentration was 0.08 mol% ErF_3_, due to the evaporation of the substance during the growth process. The crystals were grown in our Bridgman equipment using a shaped graphite furnace [23]. The crystals were obtained in vacuum (~10^−1^ Pa) using a spectral pure graphite crucible, with a pulling rate of 4 mm h^−1^. More details about the growth conditions are described in [7,9]. The obtained crystals were transparent, ~10 mm in diameter and ~5 cm in length, free of visible inclusions or cracks (Figure 1). In order to investigate the spectroscopic properties, the crystals were cleaved from the bottom to the top into 12–17 slices with a thickness of 2.5 mm. In order to study the influence of the ErF_3_ concentration on the optical absorption and emission spectra, we chose a slice from each crystal, cleaved approximately in the middle of the crystal (see Figure 1a,b,d). Some characteristics of the chosen slices are described in Table 1. 

**Figure 2:**
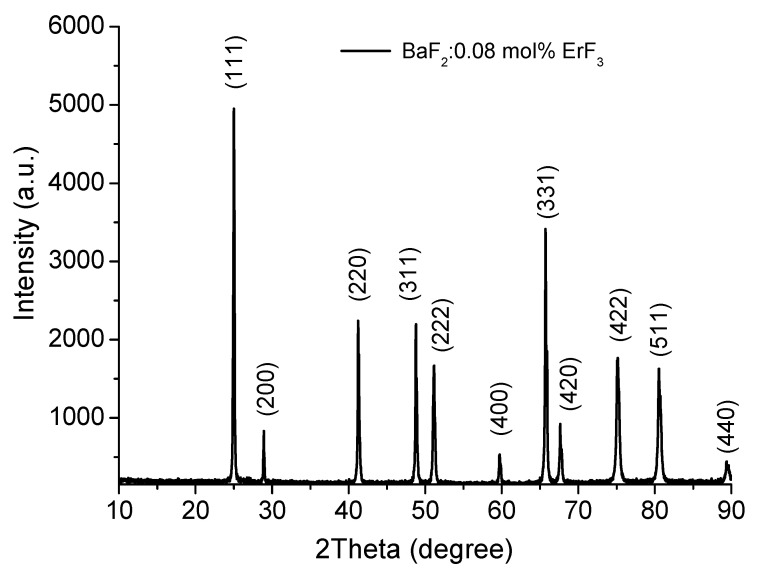
XRD pattern of BaF_2_: 0.08 mol% ErF_3_ sample.

The room temperature optical absorption spectra in the 250–850 nm range were recorded using a Shimadzu 1650 PC spectrophotometer. The spectrophotometer uses an automatic correction for baseline correction. The correction subtracts the absorbance value at a specific wavelength from all wavelengths across the sample spectrum. The correction takes into account the effect of instrument noise and the light scattering due to the possible undesired particles in the sample. In order to measure the room temperature luminescence spectra in the UV-VIS domain, a FLS 980–Edinburgh Instruments spectrofluorometer was used. Stationary and time-resolved photoluminescence measurements, with a scan slit of 0.1 nm, were taken. The excitation source was an Xe lamp for CW measurements, and for photoluminescence kinetics measurements, the pulsed microseconds Xe flash-lamp µF2 and nanoseconds flash lamp nF920 was used. For stationary and time-resolved measurements, the PMT Hamamatsu R928P detector was used. To check the crystalline structure, XRD analysis was performed using an X-ray diffractometer (PW 3040/60 X’Pert PRO) with Cu-Kα radiation (λ = 1.5418 Å). Figure 2 shows the XRD pattern for the BaF_2_: 0.08 mol% ErF_3_-grinded crystal sample. The diffraction peaks correspond to the cubic phase according to ICDD Cards No 00-004-0452, No 01-085-1341 and No 00-002-1157, and in good agreement with the published data of Bitam et al. [16]. No additional peaks that can be associated with undesired impurities were observed. The crystalline planes corresponding to the peaks are shown in Figure 2. The obtained lattice parameters are a = b = c = 6.2065 Å, Fm3m space group, α = β = γ = 90°.

The radiative decay time was calculated using the instrument software F980 reconvolution fit function, taking in account the instrumental contribution IRF to the decay curve. The branching ratios, the emission transition probabilities and the radiative lifetimes were obtained using the Judd–Ofelt (JO) model [20,21]. The influence of Er^3+^ ion concentration on the JO parameters and on the radiative lifetime was also investigated. 

## 3. Results

### 3.1. Optical Absorption Spectra 

**Figure 3:**
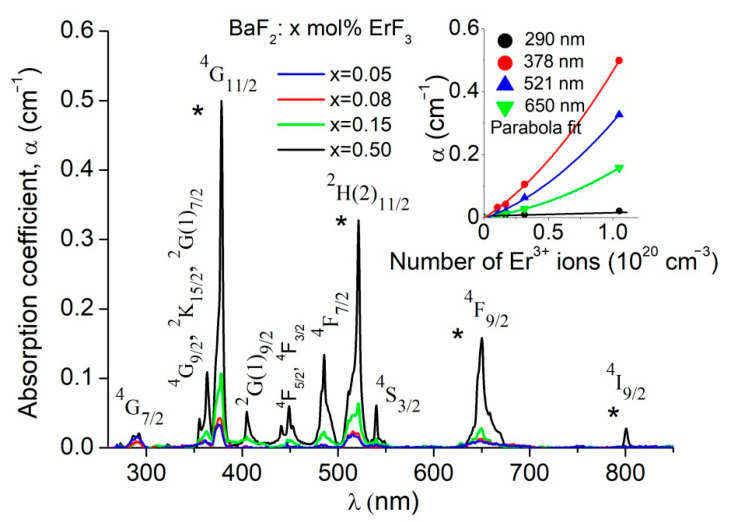
Optical absorption spectra of BaF_2_: x mol% ErF_3_ crystals. The insert shows the influence of the Er^3+^ ion number on the intensity of the main absorption bands. The asterisks indicate the absorption bands used for Judd–Ofelt (JO) analysis.

In order to study the influence of the ErF_3_ concentration on the optical absorption spectra, we eliminated the different backgrounds of the samples. The optical absorption spectra of the ErF_3_:BaF_2_ samples (indicated in Table 1) are shown in Figure 3. In the 250–850 nm domain, the absorption spectra consist of 10 absorption bands. The absorption bands correspond to the transitions from the ^4^I_15/2_ ground state to the Er^3+^ ions excited states, specified in the figure.

The absorption bands are broad and structured. Due to the charge compensation process, the energy levels of the Er^3+^ ions split causing the formation of broad and structured absorption bands. The most intense absorption bands peak at 378.5 nm, 521 nm and 650 nm. The intensity (the absorption coefficient, α) of these bands does not increase linearly with the ErF_3_ concentration (respectively, the number of ions, N, in the host), but parabolically (see the insert in Figure 3). The asterisks in the figure indicate the bands used in the JO analysis.

### 3.2. Judd–Ofelt Analysis

Information about the luminescence properties of rare-earth-doped fluoride can be obtained using the Judd–Ofelt model [20,21]. This approximation permits the determination of the transition probabilities using only the optical absorption spectra [10,20,21]. In order to calculate the JO intensity parameters Ω_2_, Ω_4_ and Ω_6_, we have used a set of four absorption bands (indicated by asterisk in Figure 3). These bands correspond to the transitions: ^4^1_15/2_—^4^I_9/2_ (802 nm), ^4^1_15/2_—^4^F_9/2_ (653 nm), ^4^1_15/2_—^2^H(2)_11/2_ (519 nm) and ^4^1_15/2_—^4^G_11/2_ (377 nm). The experimental line strength, *S_meas_*, is obtained from the absorption spectra by calculating the absorption band area (*Σ*) (see Table 2).

**Figure 4:**
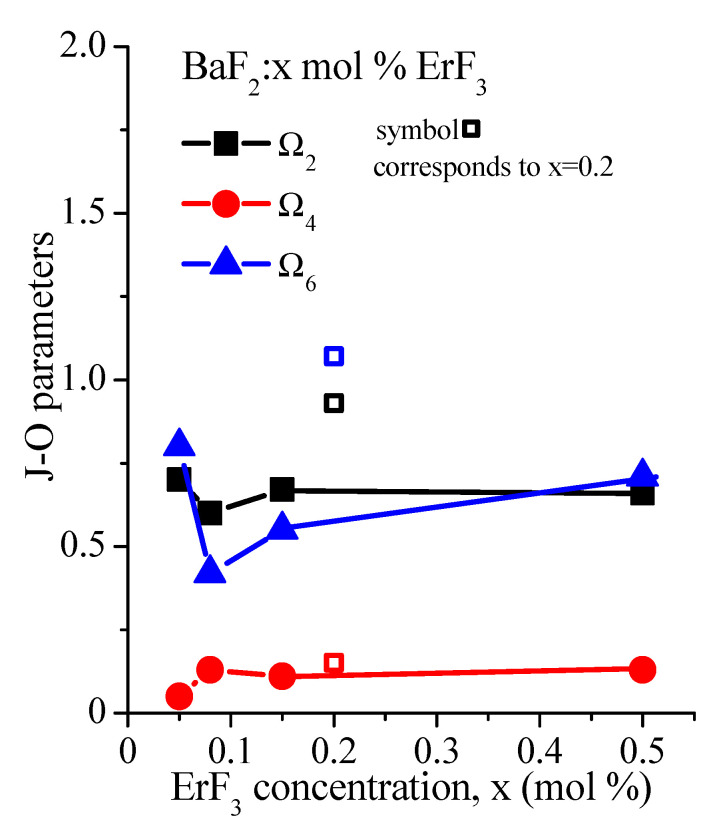
Influence of the ErF_3_ concentration on the Judd–Ofelt parameters, Ω_t_ (10^−20^ cm^2^). The values corresponding to the concentration of 0.2 mol% ErF_3_ are designated by open symbols and come from paper [9].

In order to obtain the JO parameters Ω_i_ (i = 2, 4, 6) and the measured (experimental) line strength, we solved a set of four equations corresponding to the four transitions under study. These calculations were made for the four ErF_3_ concentration samples using the Levenberg–Marquardt algorithm. The influence of the ErF_3_ concentration on the obtained JO parameters is shown in Table 3 and Figure 4. The spectroscopic quality factor χ is also given. Significant errors usually occur in the estimation of JO parameters because it is difficult to obtain accurate absorption line strengths in the case of broad and structured absorption bands (as in our case), and due to the JO model itself [9].

The calculated line strength is $S_{\mathrm{calc}}=S_{JJ^{\prime }}^{\mathrm{ed}}+S_{JJ^{\prime }}^{\mathrm{md}}$ where $S_{JJ^{\prime }}^{\mathrm{ed}}$ is the electric dipole (ed) line strength and $S_{JJ^{\prime }}^{\mathrm{md}}$ is the contribution of the magnetic dipole (md) transition. These line strengths were calculated using JO parameters, and the values of the reduced matrix elements for the chosen Er^3+^ bands from those tabulated in the work of Kaminskii [10]. The measured and calculated absorption line strengths for transitions ^4^I_15/2_ → [^4^I_9/2_; ^4^F_9/2_; ^2^H(2)_11/2_; ^4^G_11/2_] are shown in Table 4. The root-mean-square deviation, defined by $\Delta S_{\mathrm{rms}}={\left[{\left(q-p\right)}^{-1}\sum {\left(S_{\mathrm{calc}}-S_{\mathrm{meas}}\right)}^{2}\right]}^{1/2}$ is a measure of the accuracy of the fit; *q* is the number of analyzed spectral bands (*q* = 4) and *p* is the number of the parameters sought (*p* = 3). The obtained values for the root-mean-square (r.m.s.) deviation are shown in Table 4.

In order to calculate the radiative lifetime *(*τ_rad_) for an excited state *J*, we used the relationship $\tau_{\mathrm{rad}}=1/\sum A_{JJ^{\prime }}$, where *A_JJ′_* is the spontaneous emission probability and the sum is taken over all final lower-lying states *J’*. The fluorescence branching ratio was estimated using the relationship $\beta_{JJ^{\prime }}=\tau_{\mathrm{rad}}A_{JJ^{\prime }}$. The value of the radiative emission probabilities, the branching ratios and the radiative lifetimes are given in Table 5.

A comparison of the calculated radiative lifetimes (τ_rad_) and those measured by other authors is shown in Table 6.

The discrepancy between the calculated and measured lifetimes by other authors can indicate the existence of an energy migration, thermal coupling between manifolds and/or strong emission reabsorption that cannot be described using the JO model.

### 3.3. Photoluminescence and PL Kinetics

**Figure 5:**
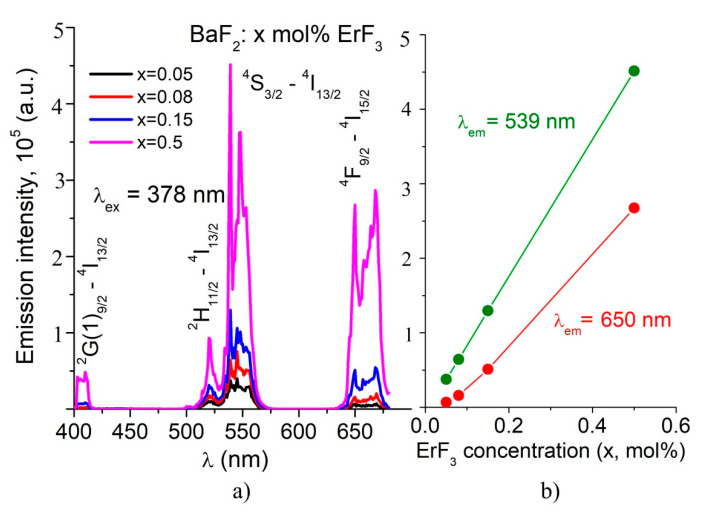
(**a**) Room temperature emission spectra of BaF_2_: x mol% ErF_3_ samples by λ_exc_ = 378 nm excitation. (**b**) Influence of the ErF_3_ concentration on the PL intensity of the green and red emissions.

**Figure 6:**
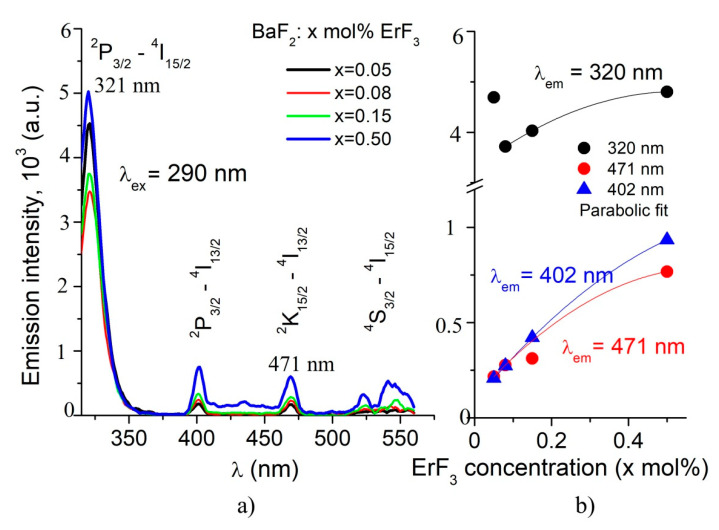
(**a**) Room temperature emission spectra of BaF_2_: x mol% ErF_3_ samples by λ_exc_ = 290 nm excitation. (**b**) Influence of the ErF_3_ concentration on the PL intensity of the UV emission (321 nm) and of the less intense emissions, at 402 nm and 471 nm.

In order to obtain the room temperature emission spectra, two absorption bands were used for excitation, namely λ_exc._ = 378 nm (^4^I_15/2_ → ^4^G_11/2_ transition) and λ_exc_. = 290 nm (^4^I_15/2_ → ^4^G_7/2_ transition). The emission spectra of the studied samples are shown in Figure 5 and Figure 6. By excitation at 378 nm, we obtained three broad emission bands (Figure 5). The red band, around 660 nm, has two peaks at 650 nm and 668 nm. The green band is broad, with three peaks at 547 nm, 539 nm and 521 nm. The less intense blue band is centered at 410 nm.

By excitation at 290 nm, the emission spectra are characterized by five photoluminescence bands: four weak emissions at 403 nm, 471 nm, 523 nm and 539 nm, and a very strong emission at 321 nm (Figure 6). The UV emission reported in our previous work corresponds to the 0.2 mol% ErF_3_ sample [9]. As the ErF_3_ concentration increases, the intensity of the emission bands increases (see Figure 5b and Figure 6b).

**Figure 7:**
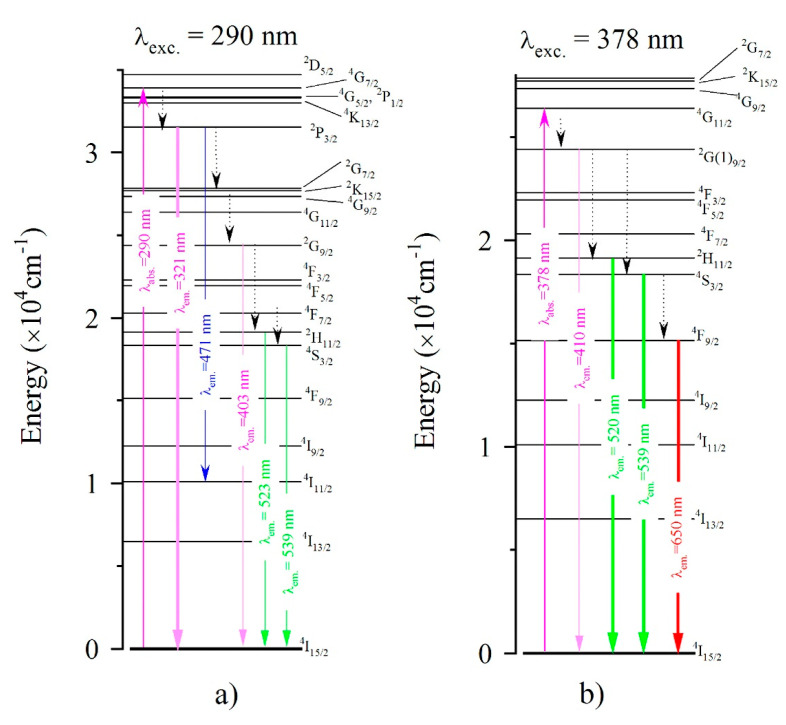
The energy-level diagram of Er^3+^ ions. The emission bands: (**a**) under excitation at 290 nm and (**b**) at 380 nm, are also shown.

Taking into account the optical absorption spectra and the emission spectra, in Figure 7 we show the energy level diagram of the Er^3+^ ion. The emission bands, under excitation at 290 nm and 378 nm, are also shown.

**Figure 8:**
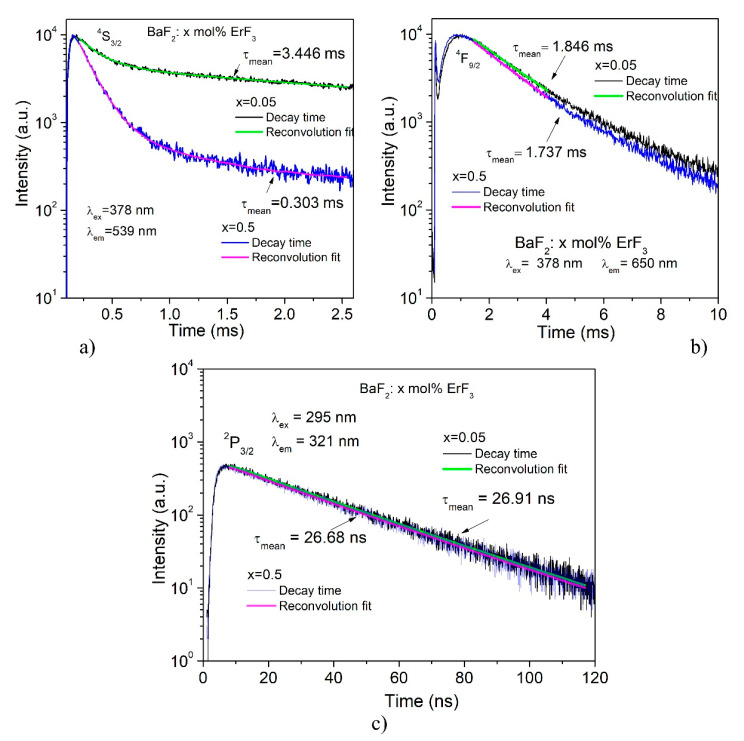
(**a**) The decay curves of the ^4^S_3/2_ → ^4^I_15/2_ transition (green emission, 539 nm) by excitation at 378 nm for two ErF_3_ concentrations (0.05 and 0.5 mol%). (**b**) The decay curves of the ^4^F_9/2_ → ^4^I_15/2_ transition (red emission, 650 nm) by excitation at 378 nm for two ErF_3_ concentrations (0.05 and 0.5 mol%). (**c**) The decay curves of the ^2^P_3/2_ → ^4^I_15/2_ transition (UV emission, 321 nm) by excitation at 295 nm for two ErF_3_ concentrations (0.05 and 0.5 mol%).

The time-resolved PL measurements give information about the decay times. Figure 8a,b shows the decay curves for the green emission (^4^S_3/2_ → ^4^I_15/2_ transition) and for the red emission (^4^F_9/2_ → ^4^I_15/2_ transition) for two ErF_3_ concentrations (0.05 and 0.5 mol%) in BaF_2_. The decay curves of all studied concentrations are given in the insert of the figure. The decay times corresponding to these emissions demonstrate non-mono exponential behavior and therefore it was fitted with a double exponential function. The decay curves of the ^2^P_3/2_ → ^4^I_15/2_ transition (UV emission, 321 nm) by excitation at 295 nm for two ErF_3_ concentrations (0.05 and 0.5 mol%) are shown in Figure 8c.

The mean decay times, τ_mean__,_ for ^4^S_3/2_, ^4^F_9/2_ manifolds were calculated using Equation [24]:

$$\tau_{\mathrm{mean}}=\frac{A_{1}\tau_{1}^{2}+A_{2}\tau_{2}^{2}}{A_{1}\tau_{1}+A_{2}\tau_{2}}$$

where *τ_1_* and *τ_2_* are the radiative decays of the non-mono exponential fitting curve.

The obtained values of mean decay times are shown in Table 7.

The PL decay times of the green and red emissions by excitation at 378 nm are of the order of *ms* and vary with the Er^3+^ ion concentration, while the decay times for the emissions obtained by 290 nm excitation are of the order of *ns* and depend slightly on the Er^3+^ ion concentration. These values are comparable to those obtained (25 ns) by Yang et al. [25] for Ce:BaF_2_ for emission at 324 nm by 291 nm excitation.

## 4. Discussion

When trivalent ions (Er^3+^ ions in our case) are dissolved in BaF_2_, the Er^3+^ ions replace the Ba^2+^ ions in the lattice. The charge compensation process takes place in order to maintain the neutrality of the system. This process is performed by placing the interstitial fluorine ions in different positions relative to the Er^3+^ ions. At very low RE concentrations (<0.01), only isolated centers are created, namely centers with cubic (*O_h_*), tetragonal (*C_4v_*) and trigonal (*C_3v_*) site symmetry. As concentration of the trivalent ions increases, in addition to isolated centers, various aggregates (clusters) are created [2,3,26,27]. The ten optical absorption bands, shown in Figure 3, correspond to the transitions from the ground state (^4^I_15/2_) to the excited states of the Er^3+^ ions. The absorption bands are broad and structured due to the various isolated centers and clusters created by the charge compensation effect. In lightly doped BaF_2_:ErF_3_ crystals, the dominant isolated center has C_3v_ symmetry, as shown by Wells et al. [3]. Using the Gaussian multi-peaks decomposition, for the asterisk-specified bands in Figure 3, we obtained the following major peaks: 372 nm and 378 nm, 511 nm and 521 nm and 643 nm and 648 nm, respectively. We assigned the 372 nm, 511 nm and 643 nm peaks to the *C_3v_* (*NNN*) site, and the 378 nm, 521 nm and 648 nm peaks to clusters (aggregates) [28].

**Figure 9:**
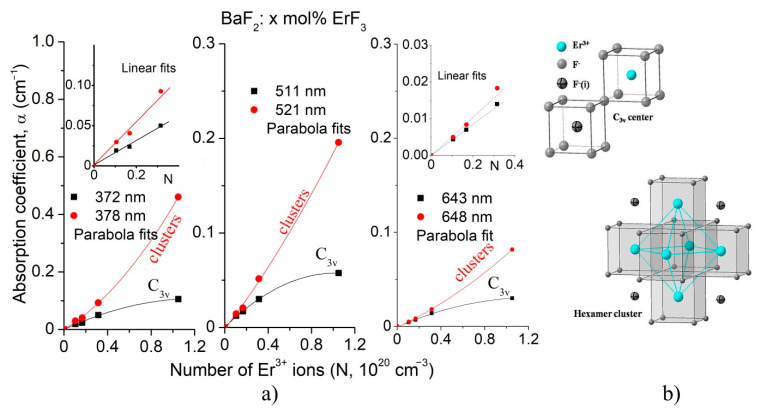
(**a**) Influence of the ErF_3_ concentration on the intensity of the components of the three absorption bands specified by asterisk in Figure 3. (**b**) The sketches of the C_3v_ center and hexamer cluster.

The influence of ErF_3_ concentration (or the number of Er^3+^ ions in the samples) on the intensity of these components is shown in Figure 9a. Overall, the intensity (the absorption coefficient, α) of these bands does not increase linearly with the number of Er^3+^ ions (*N*) in the BaF_2_, but parabolically. Up to relatively low ErF_3_ concentrations (~0.15 mol% ErF_3_ or 0.3 × 10^20^ cm^−3^ ions), both the intensity of the peaks associated with the C_3v_ site and of the clusters increases linearly with ErF_3_ concentration, as presented in the inserts in Figure 9a. This behavior is normal because, at low ErF_3_ concentrations, the probability of creating both isolated centers (C_3v_) and clusters is approximately the same. As the concentration increases, much fewer C_3v_ centers are created than clusters. According to Beer’s law, the absorption coefficient is proportional to the number of absorbent centers. As a result, the peak intensity corresponding to the C_3v_ component (372 nm) will tend to saturate, while the intensity corresponding to the clusters (378 nm) will increase much more. Overall, the behavior shows a parabolic aspect (but of two kinds), as seen in Figure 9a. Other authors [3,4,6,29] also reported this behavior.

The majority of studies regarding luminescence RE ion-doped BaF_2 v_ refer to emissions obtained mainly by pumping in the IR domain [14,15,16]. Comparing the emission spectra of our samples by excitation at λ_ex_ = 290 nm and λ_ex_ = 380 nm, it was concluded that the emission at 321 nm is the most intense (see Figure 5a and Figure 6a).

The emission spectra by excitation at 380 nm consist on three bands, one very weak blue band at about 405 nm, and the well-known green and red emissions (Figure 5a). These emissions are due to the transitions from ^2^G(1)_9/2_, ^4^S_3/2_ and ^4^F_9/2_ excited levels to the ^4^I_15/2_ ground level. The intensity of the green and red emissions are comparable; the green emission is ten times more intense than the blue emission. As ErF_3_ concentration increases, the intensities of the emission bands increase linearly (see Figure 5b). Other authors also reported emissions under excitation at 378 nm in a BaF_2_ host [15,16].

**Figure 10:**
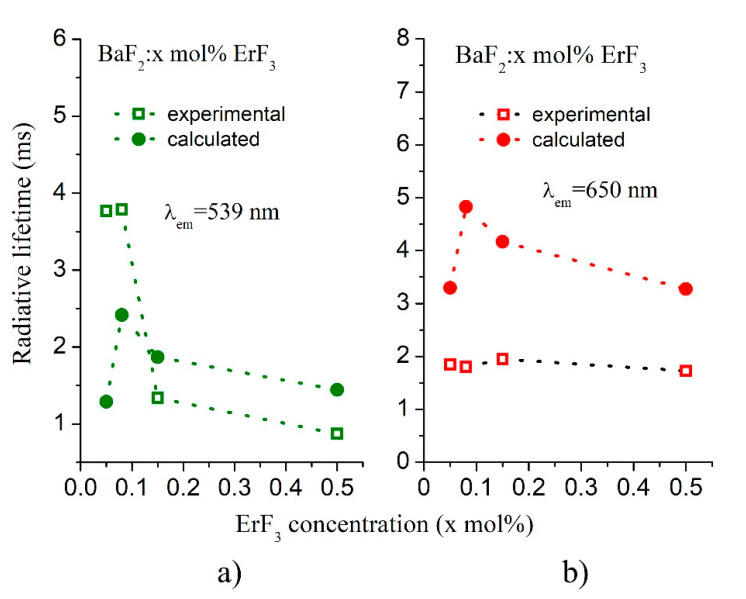
Influence of ErF_3_ concentration on the experimental and calculated radiative lifetime for (**a**) the green emission and (**b**) the red emission.

The influence of ErF_3_ concentration on the experimental and calculated radiative lifetime for the green emission and for the red emission is shown in Figure 10. The values of the calculated radiative lifetime are higher than the values found experimentally. The difference between the calculated lifetime and the experimentally measured lifetime is due to the errors with which the JO parameters are calculated.

**Figure 11:**
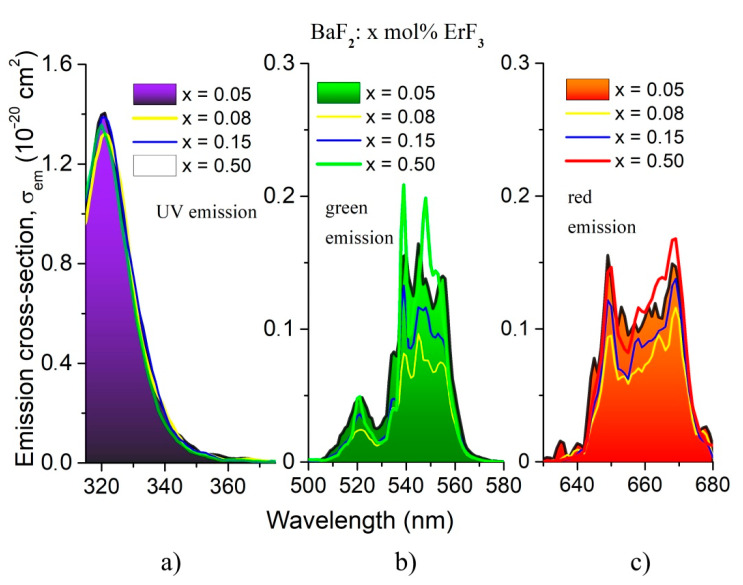
Emission cross-sections of BaF_2:_ x mol% ErF_3_ crystals: (**a**) the UV emission; (**b**) the green emission; and (**c**) the red emission. The UV, green and red emissions of the 0.05 mol% ErF_3_ sample are highlighted.

In order to estimate the emission cross-section, corresponding to the observed emissions, the Füchtbauer–Ladenburg relationship [30] was applied:

$$\sigma_{em}\left(\lambda \right)=\frac{\lambda^{5}I\left(\lambda \right)}{8\pi {\left[n\left(\lambda \right)\right]}^{2}c\tau_{\mathrm{mean}}\int \lambda I\left(\lambda \right)d\lambda }$$

where *I*(*λ*) is the emission intensity at each wavelength, τ_mean_ is the mean radiative lifetime of the upper laser level and *β* is the branching ratio, *n* is the refractive index and *c* is the velocity of light. The UV, green and red emissions cross-sections are shown in Figure 11.

To evaluate the laser performance of our samples, the optical gain parameter $G=\sigma_{\mathrm{em}}\tau_{\mathrm{mean}}$, was calculated for every sample. The obtained values are given in Table 8.

The optical gain parameter varies with the ErF_3_ concentration for the green and red emissions, while for the UV emission, it does not vary. The highest value is obtained for the 0.05 mol% ErF_3_ concentration sample; therefore, this concentration should be more efficient as laser material in comparison with the other concentrations. The quantum efficiency for UV emission varies between 2.9% and 6.4%, and in the case of the red emission, it varies between 37.5% and 56%.

By excitation at 290 nm, the emission spectra reveal four weak emission bands in the visible domain and one band in UV, five times more intense than those in the visible domain (Figure 6a). The less intensive emissions peak at 403 nm, 471 nm, 523 nm and 539 nm. The intensities of these visible field emissions are five times weaker than those obtained by 378 nm excitation. The intensity of the UV emission (321 nm) is comparable with the intensity of the green emission obtained by 378 nm excitation. The intensities of the emissions vary parabolically with the ErF_3_ concentration (Figure 6b). This emission probably includes the self-trapped exciton (STE) component that involves non-linear behavior regarding Er^3+^ ion concentration in comparison to the red and green emissions pumped at 378 nm.

**Figure 12:**
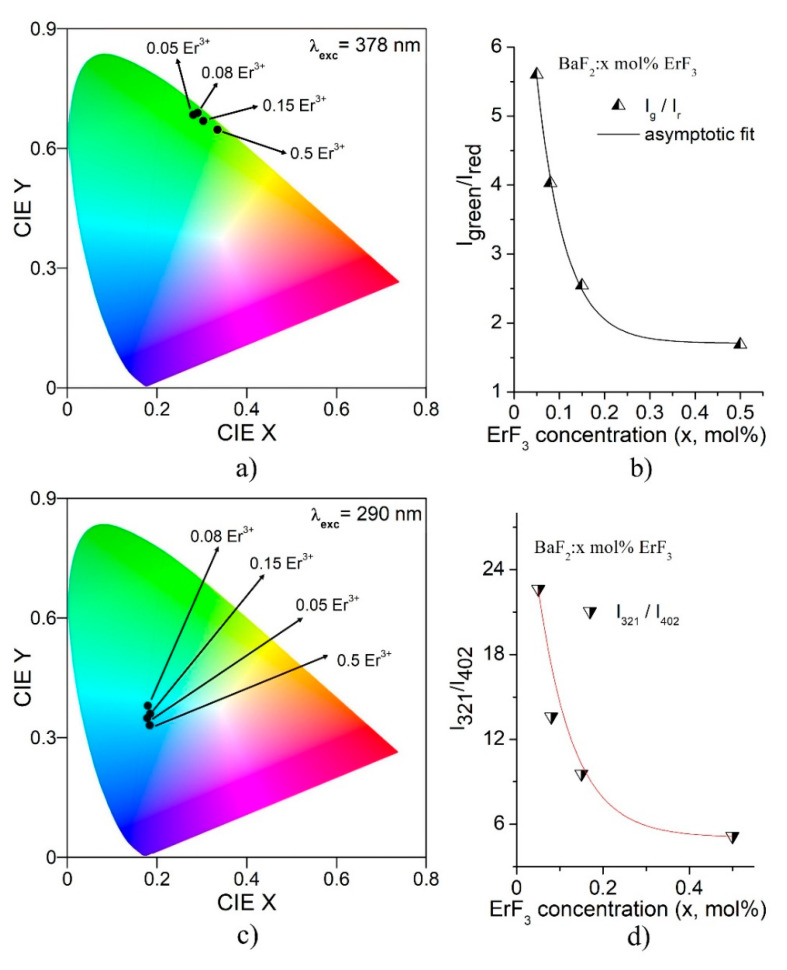
CIE coordinates for the emissions of BaF_2_: x mol% ErF_3_ samples. (**a**) For emissions obtained at 378 nm excitation; (**b**) ErF_3_ concentration dependence of the green to red intensity ratio (I_g_/I_r_); (**c**) CIE coordinates for emissions obtained at 290 nm excitation; (**d**) ErF_3_ concentration dependence of the 321 nm to 402 nm intensity ratio (I_321_/I_402_).

The International Commission on Illumination (CIE) charts for all the samples, for the emissions obtained under excitation at 378 nm and 290 nm, are shown in Figure 12a,c, respectively. The CIE coordinates were obtained using Gocie V2 software [31]. The CIE 1931 color coordinates are as follows. For the excitation at 378 nm, the color coordinates are: (X = 0.28, Y = 0.68) for 0.05 mol% Er^3+^, (X = 0.29, Y = 0.69) for 0.08 mol% Er^3+^, (X = 0.30, Y = 0.67) for 0.15 mol% Er^3+^ and (X = 0.33, Y = 0.65) for 0.5 mol% Er^3+^. For the excitation at 291 nm, the color coordinates are: (X = 0.18, Y = 0.35) for 0.05 mol% Er^3+^, (X = 0.18, Y = 0.38) for 0.08 mol% Er^3+^, (X = 0.18, Y = 0.36) for 0.15 mol% Er^3+^ and (X = 0.18, Y = 0.33) for 0.5 mol% Er^3+^. In the case of the emissions by 378 nm excitation, the ratio between the green (539 nm) intensity and the red (650 nm) intensity decreases from 5.6 to 1.7 (three times), as the ErF_3_ concentration increases (see Figure 12b). According to Figure 12a,b, the green emission is strong for concentrations up to ~0.2 mol% ErF_3_, therefore the green color dominates in these cases. For higher concentrations, the intensity of the red emission increases and the color moves to the yellow domain of the chart. In the case of the emissions obtained by 290 nm excitation, the ratio between the UV (321 nm) intensity and the violet (402 nm) intensity decreases four times, as the ErF_3_ concentration increases (see Figure 12d). For the ErF_3_ concentration higher than ~0.2 mol%, the intensity of the violet emission increases and the color moves a little to the blue region of the chart. Therefore, the blue color dominates.

The emission intensity corresponding to the lowest concentration (0.05 mol%) is comparable to that obtained for the 10 times higher concentration (0.5 mol%). The influence of the ErF_3_ concentration on this emission has not been reported previously. The emission at 321 nm is attributed to transition ^2^P_3/2_ → ^4^I_15/2_. The UV emission at 314 nm, corresponding to the ^4^D_5/2_ → ^4^I_13/2_ transition, was observed in Er:CaF_2_ crystals [22]. The emission at ~321 nm was observed in similar crystals using various excitation techniques, including thermo, radio, X-ray and photoluminescence [14,32,33,34]. Wojtowicz et al. [14,34] attributes this emission to the self-trapped exciton STE emission; they observed this emission when pumped in VUV range, near the band-gap wavelengths. Several concurring pumping channels were also observed.

When the ^4^G_7/2_ energy level (λ—290 nm) was pumped, the Er-bond exciton emission (at 321 nm) took place from the ^2^P_3/2_ manifold. As mentioned by Wojtowicz et al. [14], because the ^2^P_3/2_ level is in the middle of the STE emission band, the energy transfer from the STE to the Er^3+^ ion becomes effective. This could be the cause of the weak photoluminescence at 403 nm, 471 nm, 523 nm and 539 nm (Figure 6a). The STE component emission at 321 nm involves the ^2^P_3/2_ → ^4^I_15/2_ transition. This mechanism probably involves the emission at 471 nm, which can be attributed to transition ^2^P_3/2_ → ^4^I_11/2_ or to ^4^F_7/2_→^4^I_15/2_. The less intensive emissions at 403 nm, 523 nm and 539 nm can be attributed to the ^2^G(1)_9/2_ → ^4^I_15/2_ and ^2^H_11/2_, ^4^S_3/2_ → ^4^I_15/2_ transitions, respectively.

## 5. Conclusions

The optical and luminescence behavior of BaF_2_ crystals doped with ErF_3_ was investigated. The crystals were obtained by the Bridgman method. As the ErF_3_ concentration increases, the intensity of the absorption peaks corresponding to the clusters also increases, but stronger than the peaks attributed to the *C_3v_* site. In order to obtain the emission transition probabilities, branching ratios, radiative lifetimes and the gain parameter, the Judd–Ofelt approximation was used. The obtained parameters were compared with the measured values and those reported in the literature. The emission spectra obtained by 378 nm excitation reveal two major bands, the green (539 nm) and the red (650 nm) emissions. The highest value (6.8 × 10^−24^ cm^2^·s) of the estimated gain parameter was obtained for the green emission for the 0.05 mol% ErF_3_ sample. For the red emission, the highest gain parameter was 3 × 10^−24^ cm^2^·s. Therefore, this concentration should be more efficient for laser applications than the other concentrations. The quantum efficiency varies between 37.5% and 56%. Under excitation at 290 nm, along with the weak green and red emissions, a new, strong UV band (321 nm) was obtained. The gain parameter for the UV emission is an order of a magnitude smaller (~0.3 × 10^−24^ cm^2^·s) than in the case of the green and red emissions obtained at 378 nm excitation. The gain parameter for the UV emission does not vary with the ErF_3_ concentration. The quantum efficiency varies between 2.9% and 6.4%. The influence of ErF_3_ concentration on the Judd–Ofelt parameters and on the luminescence of Er:BaF_2_ crystals has not been reported in the exiting literature.

## Figures and Tables

**Table 1:** Slice parameters. The cleavage plane is (111).

BaF_2_: x mol% ErF_3_		
x	*d* (mm) Slice Thickness	*N* (10^20^ cm^−3^) Er^3+^ Ion Concentration
0.05 (*Slice* 8)	2.21	0.105
0.08 (*Slice* 7)	2.32	0.168
0.15 (*Slice* 9)	2.37	0.315
0.50 (*Slice* 9)	2.62	1.050

**Table 2:** The mean wavelength, the wavelength range and the integrated absorption cross-section, ∑, of the selected absorption peaks.

Transition ^4^I_15/2_ ↓	λ_mean_ (nm)	Wavelength Range (nm)	$\sum =\int \sigma \left(\lambda \right)d\lambda \;(10^{-20}\;{\mathrm{cm}}^{2}\cdot \mathrm{nm})$			
			BaF_3_: x mol% ErF_3_			
			x = 0.05	0.08	0.15	0.50
^4^I_9/2_	802	788–808	0.001	0.003	0.005	0.023
^4^F_9/2_	653	619–680	0.020	0.023	0.051	0.066
^2^H(2)_11/2_	519	502–532	0.025	0.033	0.071	0.086
^4^G_11/2_	377	369–386	0.022	0.035	0.072	0.089

**Table 3:** The influence of ErF_3_ concentration on the Judd–Ofelt parameters and on the spectroscopic quality factor (χ), including estimated errors of JO parameters.

Ω_i_ (10^−20^ cm^2^)	ErF_3_ Concentration (mol%)			
	0.05	0.08	0.15	0.50
Ω_2_	0.7083 ± 0.31	0.6073 ± 0.50	0.6749 ± 0.34	0.6678 ± 0.51
Ω_4_	0.0495 ± 0.02	0.1397 ± 0.03	0.1118 ± 0.02	0.1376 ± 0.037
Ω_6_	0.8036 ± 0.05	0.4271 ± 0.08	0.5525 ± 0.06	0.7152 ± 0.08
Χ = Ω_4_/Ω_6_	0.06163	0.32717	0.20232	0.19237

**Table 4:** Measured and calculated absorption line strengths (10^−22^ cm^2^) for the four samples.

BaF_3_: x mol% ErF_3_	x=	0.05		0.08		0.15		0.50	
^4^I_15/2_ ↓ Transition	λ (nm)	$S_{DE}^{meas}\;$	$S_{DE}^{calc}$	$S_{DE}^{meas}$	$S_{DE}^{calc}$	$S_{DE}^{meas}$	$S_{DE}^{calc}$	$S_{DE}^{meas}$	$S_{DE}^{calc}$
^4^I_9/2_	802	1.62	1.65	2.89	2.84	2.51	2.48	3.14	3.09
^4^F_9/2_	653	39.7	39.76	27.2	27.21	31.51	31.50	40.41	40.40
^2^H(2)_11/2_	519	62.3	59.94	49.0	52.98	55.07	57.81	55.78	59.87
^4^G_11/2_	377	75.1	77.04	71.17	68.10	76.44	74.31	80.09	76.92
ΔS_rms_ [×10^–20^ cm^2^]		0.031		0.050		0.035		0.052	

**Table 5:** The radiative emission probabilities (*A_JJ’_*), the branching ratios (*β*) and the radiative lifetimes (τ_rad_).

		BaF_2_: x mol% ErF_3_											
Transitions	*λ*_mean_ (nm)	x = 0.05			x = 0.08			x = 0.15			x = 0.5		
		$A_{JJ^{\prime }}$ (s^−1^)	*β*	τ_rad_ (ms)	$A_{JJ^{\prime }}$ (s^−1^)	*β*	τ_rad_ (ms)	$A_{JJ^{\prime }}$ (s^−1^)	*β*	τ_rad_ (ms)	$A_{JJ^{\prime }}$ (s^−1^)	*β*	τ_rad_ (ms)
^4^I_13/2_ → ^4^I_15/2_	1522	71.9	1	13.9	52.5	1	19.1	59	1	16.9	67.6	1	14.8
^4^I_11/2_ → ^4^I_15/2_ →^4^I_13/2_	976 2778	54.4 6.5	0.89 0.11	16.4	30.0 3.6	0.89 0.11	29.8	38.2 4.6	0.89 0.11	23.3	48.6 5.9	0.89 0.11	18.4
^4^I_9/2_ → ^4^I_15/2_ →^4^I_13/2_ →^4^I_11/2_	802 1739 4651	5.7 20.40.2	0.22 0.78 ~0	38	9.85 10.9 0.12	0.47 0.52 ~0	47.9	8.6 14.1 0.1	0.38 0.62 ~0	43.9	10.71 18.2 0.2	0.37 0.63 ~0	34.4
^4^F_9/2_ → ^4^I_15/2_ →^4^I_13/2_ →^4^I_11/2_ →^4^I_9/2_	653 1156 1980 3448	261.5 8.7 30.5 2.6	0.86 0.03 0.1 ~0	3.3	178.9 7.1 18.8 2.5	0.86 0.03 0.09 0.01	4.8	207.2 7.7 22.3 2.5	0.86 0.03 0.09 0.01	4.2	265.7 9.6 27.7 2.6	0.87 0.03 0.09 0.01	3.3
^4^S_3/2_ → ^4^I_15/2_ →^4^I_13/2_ →^4^I_11/2_ →^4^I_9/2_ →^4^F_9/2_	543 844 1212 1639.4 3125	521.5 217.7 15.7 22 0.3	0.67 0.28 0.02 0.03 ~0	1.3	277.2 115.7 8.5 12.6 0.2	0.67 0.28 0.02 0.03 ~0	2.4	358.6 149.6 10.9 15.8 0.2	0.67 0.28 0.02 0.03 ~0	1.9	464.2 193.7 14.1 20.4 0.3	0.67 0.28 0.02 0.03 ~0	1.4
^2^H(2)_11/2_ → ^4^I_15/2_ →^4^I_13/2_ →^4^I_11/2_ →^4^I_9/2_ →^4^F_9/2_ →^4^S_3/2_	519 791 1105 1449 2500 12,500	668.5 66.9 13.4 19.9 2.7 ~0	0.87 0.09 0.02 0.03 ~0 ~0	1.3	590.9 61.6 12.8 13.7 2.3 ~0	0.87 0.09 0.02 0.02 ~0 ~0	1.5	644.8 63.7 13.2 16.2 2.6 ~0	0.87 0.09 0.02 0.02 ~0 ~0	1.4	667.7 66.8 14.2 18.5 2.5 ~0	0.87 0.09 0.02 0.02 ~0 ~0	1.3
^4^F_7/2_ → ^4^I_15/2_ →^4^I_13/2_ →^4^I_11/2_ →^4^I_9/2_ →^4^F_9/2_ →^4^S_3/2_ →^2^H(2)_11/2_	486 725 980 1242 1942 5128 8696	1016.6 10.4 34.2 52.6 9.5 ~0 0.1	0.9 ~0 0.03 0.05 ~0 ~0 ~0	0.9	573.5 29.2 25.9 33.7 9.3 ~0 0.1	0.85 0.04 0.04 0.05 0.01 ~0 ~0	1.5	721.7 23.4 28.9 40.1 9.4 ~0 ~0	0.88 0.03 0.03 0.05 0.01 ~0 ~0	1.2	932.1 28.7 36.7 48.9 9.5 ~0 ~0	0.88 0.03 0.03 0.05 0.01 ~0 ~0	0.9

**Table 6:** The calculated (τ_rad_) and measured radiative lifetime (τ) by other authors.

Energy Level	*λ*_m_ (nm)	Calculated Lifetime (This Work)	ErF_3_ Concentration (mol%)				Measured Lifetime τ (ms)
			0.05	0.08	0.15	0.50	
^4^F_9/2_	653	τ_rad_ (ms)	3.297	4.826	4.164	3.273	2.21 [9]
^4^S_3/2_	539		1.287	2.415	1.869	1.443	1.1 [9], 0.56 [16] 0.88 [13]
^2^H(2)_11/2_	522		1.296	1.468	1.351	1.299	0.95 [9]
^2^P_3/2_	321		0.674	0.420	0.906	0.714	0.48 [9]

**Table 7:** Experimental lifetime values obtained using reconvolution fit.

$\tau_{\mathrm{mean}}$ Lifetime	Energy Level	*λ*_m_ [nm]	ErF_3_ Concentration (mol%)			
			0.05	0.08	0.15	0.50
(ns)	^2^P_3/2_	321	26.909	26.836	26.369	26.683
(ms)	^4^S_3/2_	539	3.446	3.288	0.736	0.303
(ms)	^4^F_9/2_	650	1.846	1.812	1.948	1.737

**Table 8:** The estimated gain parameters (*G*) for the red, green and UV emissions.

	BaF_2_: *x* mol% ErF_3_				
*G* (×10^−24^ cm^2^·s)	*x* = 0.05	*x* = 0.08	*x* = 0.15	*x* = 0.5	*x* = 0.2 [9]
UV emission	0.37	0.35	0.36	0.35	-
Green emission	6.8	3.4	2.1	4.7	3.3
Red emission	3.0	1.8	2.5	2.8	6.4

## Data Availability

The data presented in this study are available on request from the corresponding author.
